# Why (not) participate in citizen science? Motivational factors and barriers to participate in a citizen science program for malaria control in Rwanda

**DOI:** 10.1371/journal.pone.0237396

**Published:** 2020-08-24

**Authors:** Domina Asingizwe, P. Marijn Poortvliet, Constantianus J. M. Koenraadt, Arnold J. H. van Vliet, Chantal M. Ingabire, Leon Mutesa, Cees Leeuwis

**Affiliations:** 1 College of Medicine and Health Sciences, University of Rwanda, Kigali, Rwanda; 2 Strategic Communication Group, Wageningen University, Wageningen, The Netherlands; 3 Laboratory of Entomology, Wageningen University, Wageningen, The Netherlands; 4 Environmental Systems Analysis Group, Wageningen University, Wageningen, The Netherlands; 5 Knowledge, Technology and Innovation Group, Wageningen University, Wageningen, The Netherlands; University of Birmingham, UNITED KINGDOM

## Abstract

This study explores the motivational factors and barriers to participate in a citizen science program for malaria control in Rwanda. It assesses the changes in motivational factors over time and compares these factors among age and gender groups. Using a qualitative approach, this study involved 44 participants. At the initial stage, people participated in the program because of curiosity, desire to learn new things, helping others, and willingness to contribute to malaria control. As the engagement continued, other factors including ease of use of materials to report observations, the usefulness of the program, and recognition also played a crucial role in the retention of volunteers. Lack of time and information about the recruitment process, perceived low efficacy of the mosquito trap, and difficulties in collecting observations were reported as barriers to get and stay involved. Some variations in the motivational factors were observed among age and gender groups. At the initial phase, young adults and adults, as well as men and women were almost equally motivated to contribute to malaria control. For the ongoing phase, for age, the two groups were almost equally motivated by recognition of their effort. Also, the opportunity for learning was an important factor among young adults while ease of use of the materials was central for adults. For gender, the usefulness of the project, ease of use of materials, and learning opportunities were important motivational factors among women, while men were more motivated by recognition of their efforts. A framework including motivational factors and barriers at each stage of participation is presented. This framework may be used to explore motivations and barriers in future citizen science projects and might help coordinators of citizen science programs to determine whom to target, by which message, and at what stage of participation to retain volunteers in citizen science projects.

## Introduction

The involvement of the public in citizen science projects (CSPs) is currently growing significantly [1–3]. The present contribution reports on a CSP focusing on malaria control in Rwanda referred to as “a CSP for malaria control” [4] and this CSP aims to provide insights in mosquito nuisance, confirmed malaria cases, and mosquito populations in a rural setting where this type of information is readily available. An important challenge with the design and implementation of CSPs is how to involve people and retain them. In this regard, motivational factors seem to be important, but information on these motivations and barriers is limited for non ICT-based CSPs. Sometimes CSPs may turn out to be unsuccessful because they do not consider these motivational factors, thus there is a waste of resources if people start participating and then drop out afterwards [5, 6]. Therefore, to establish an effective and sustainable CSP, this study explored the motivational factors and barriers to participate in the CSP for malaria control.

Some studies that explore volunteer motivations to participate in “Information Communication and Technology (ICT)”-based CSPs have been conducted in fields as diverse as agriculture [7], biodiversity and conservation [8], astronomy [9], environment [10], and health [11]. Here, the ICT-based CSPs refer to citizen science projects that use ICT tools (for example the online platform, mobile phones, etc.) in the collection and/or submission and visualization of citizen science data. While non-ICT-based CSPs refer to projects that use, for example, paper-based forms (for example the citizen science program for malaria control reported in this study). There is a large variation in motivational factors reported in these studies. For example, the motivational factors differ by country and discipline [7]. Currently, there is still a knowledge gap regarding the array of these factors in non ICT-based CSPs. Addressing this gap is important for the improvement of recruitment procedures and, as such, may contribute to the retention of volunteers.

People’s motivations to participate in CSPs include a desire to learn new things, to help others, to establish a social network, to contribute to scientific research, to help the environment, to obtain a good reputation in the community, and to further one’s career [12–15]. While motivational factors may change over time, many studies have examined these factors at a single point in time [9, 16–18]. Only a small portion has explicitly discussed these motivational factors at different points in time, either as initial and/or as retaining motivational factors [13, 19, 20]. Given that many CSPs experience a high attrition rate across different stages of participation [1, 21], there is a need to explore motivational factors and barriers at different points in time to understand what to focus on at which point of participation to retain volunteers in CSPs.

The motivational factors for participation in CSPs may differ among different groups of people and some studies indicated associations between motivational factors and demographic factors [9, 22]. For example, Raddick, Bracey [9] compared the motivational factors among men and women and found that men were more likely to participate in a CSP because they wanted to contribute to scientific research, while women were more likely to join because of personal enjoyment associated with the project. Land-Zandstra, Devilee [10] revealed contribution to science and concern for health to be more salient motivational factors to join CSP among adult people than in young people. The reasons for participating in citizen science were also compared with the volunteers’ level of education [23]. Although the contribution to science was the most prominent motivational factor across all levels of education, curiosity to engage in science, fun, and relaxation were frequent among participants who reported being at high school [23].

While there are many motivations to participate in CSPs, volunteers may also encounter barriers or challenges that limit their participation. A marine-related project, by Martin, Smith [17] revealed that the most important barrier to participate was people’s belief about limited knowledge of species that they aim to collect. Lack of time and poor or inadequate technological infrastructure were also reported to discourage volunteers to participate in CSPs [14, 20]. When volunteers encounter challenges that interfere too much with their daily activities throughout the participation, they are more likely to cease their participation at any time and any stage of participation.

This study aimed to explore the factors that determine participation and continued participation in a CSP for malaria control. The following research questions were answered: (1) What are the motivational factors to participate in a CSP for malaria control? (2) What are barriers for getting and staying involved in a CSP for malaria control? and (3) How do motivational factors change over time and vary among age and gender?

Age and gender were chosen as key demographic characteristics of interest, also because in the study area the majority of the volunteers of the CSP for malaria control have comparable low levels of education and all of them are farmers, effectively precluding these factors to use for comparative purposes.

**Fig 1:**
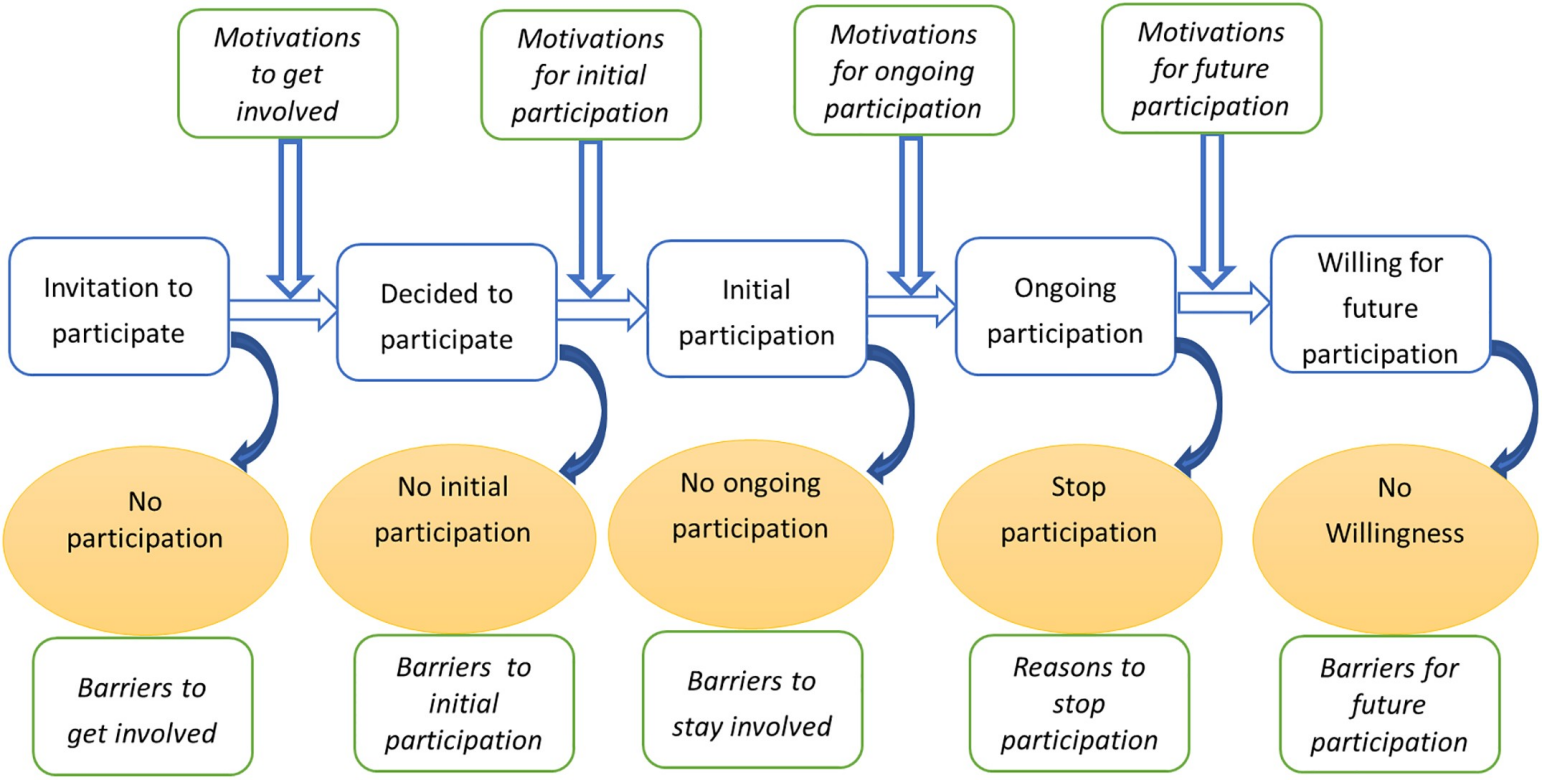
Different stages of volunteers’ participation (active collection and reporting/submission of citizen science data) in a citizen science program.

In the next section, a conceptual framework is presented that describes the different stages of volunteers’ participation in a CSP at different points in time, and different motivational factors and barriers to participation (Fig 1). This is followed by a description of the methodology of our study with a description of the CSP for malaria control. Then the results regarding the initial and ongoing participation are presented together with changes in motivational factors over time and variations among age and gender. Finally, the results are discussed in a broader context of the citizen science field.

## Conceptual framework

Volunteers’ participation in CSPs involves various stages at different points in time and these include the decision to participate or not, initial participation, ongoing participation, and future or sustained participation (Fig 1). In most of CSPs, not all those invited are willing to join. Furthermore, not all people who decide to take part in the program by registration do move to the initial participation to submit or report their first observations and stay involved [15].

Different ways have been used in the literature to categorize motivational factors [24, 25]. The categorization used most in citizen science literature is by Clary, Snyder [26] and includes six motivational functions for volunteerism: *values* (desire to help others and contribution to health and or environment), *social* (want to meet new people, socialize), *understanding* (interest in learning opportunities), *protective* (desire to address own problems), *enhancement* (want personal improvement), and *career* (interest in gaining experience) [15, 26]. These functions serve as motivation to start and continue participation in CSPs [15, 27, 28]. Another categorization of motivational factors is from Batson, Ahmad [29] who classified motivational factors in four categories: *egoism* (self-related motivations or increasing one’s welfare), *altruism* (helping others), *collectivism* (contributing to general health and environment or contributing to the overall project’s goals), and *principlism* (moral principles).

Although these authors have classified the motivational factors using different categories, it is clear that some of the categories are similar or somehow related. For example, the values function in the first categorization is related to altruism and collectivism in the second category, and different studies have used these classifications in different ways [7–9]. It is apparent that these factors may change at different stages of participation (Fig 1), and many factors may operate at one single point in time. For example, the motivations at the initial stage are subjected to change over time as citizens deepen their engagement in the project and knowledge increases [9, 29].

In the current literature, few studies have used the four categories of motivations of Batson et al. [29] to indicate motivational factors in different stages of participation in CSPs. For example, some studies indicated egoism (for example learning about bees in the Pollinator project) to be a primary motive for initial participation, while altruism and collectivism (for example, contributing to both research and environment) were associated with long term participation [8, 13]. In contrast, Land-Zandstra, Devilee [10] found contribution to research, health, and environment to be the most dominant factors that motivated people to join the Dutch iSPEX CSP, a project on measuring air quality. These differences indicate that a common framework to present these motivational factors and barriers in the different stages of participation is needed. Exploring the motivational factors and barriers in these stages provides evidence that may better inform future CSPs on what to do at each stage to encourage people to get and stay involved. Therefore, this study aimed to explore the factors that determine initial and ongoing participation in a CSP for malaria control.

## Materials and methods

### Overview of a citizen science program for malaria control in Rwanda

The study was carried out as part of a project that is being implemented in five villages of the Ruhuha sector in the Bugesera District in the Eastern province of Rwanda. Ruhuha was selected because it is a malaria-endemic area, with no current active vector surveillance. The sector is divided in five cells, and the cells are further divided in different villages. One village was randomly selected in each cell for the implementation of this CSP. On average, each village has 150 households and 45 households were invited to attend the participatory workshops where volunteers were later selected and this project was launched in 2018 [4]. A participatory workshop was used as a method to recruit volunteers in a citizen science program for malaria control. Community members collectively participated in defining the problem and identification of possible solutions with consideration of their needs [4]. Of the 185 people who attended the recruitment workshops, a total of 116 (63%) volunteers accepted to participate in the project and 69 (37%) decided not to participate.

Through co-designing [4], the goal of the project was to engage citizens in malaria control through the collection and reporting of mosquito species, mosquito nuisance experienced and confirmed malaria cases that occurred two weeks prior to reporting. When collecting mosquitoes, volunteers use a handmade trap and have to change batteries of the torches during the night. This methodology was published in a different paper [4]. Volunteers reported data on a monthly basis (collected the data every last Wednesday of the month) and the researchers provided monthly feedback. The researchers also organized dissemination workshops every four months to provide updates about the observations reported in the previous four months, offer additional knowledge on the mosquito species, as well as discuss challenges and ways forward [4]. This program was conducted for one full year (November 2018 to October 2019). Throughout the program, a high participation rate (around 93%) was observed, and variation in the participation rate was hardly noticed.

The methods are described in line with the consolidated criteria for reporting qualitative studies (COREQ) [30].

### Study design

An exploratory descriptive design with a qualitative approach was used [31]. The exploratory descriptive qualitative design is used to explain how a phenomon is manifested [31]. It allows the researcher to investigate the nature of a little understood situation or phenomon, to provide an in-depth understanding of the phenomenon under study, and allows participants to contribute to the generation of new knowledge in that particular area [31]. In the present study, the motivational factors and barriers to participate in a citizen science program for malaria control (a non-ICT-based CSP) were explored as the application of such approach in medicine and public health is very limited [32].

### Study participants

This study involved both volunteers and non-volunteers. Volunteers were defined as those who participated in the CSP on malaria control which consisted of reporting mosquito nuisance experienced or the number of confirmed malaria cases in the family, and/or participated in mosquito collection. Non-volunteers were people who only attended the participatory design workshops that were used to recruit volunteers but decided not to join the project [4]. In total, 30 volunteers across all five villages who reported mosquito nuisance and or collected mosquitoes were purposively selected to participate in this study of citizen science implementation; that is, six volunteers per village. These included both men and women of all ages (young adult: aged 23 to 35; adult: aged above 35). In addition, 14 participants who attended the participatory design workshops but did not join the project were also selected purposively. Initially, 15 non-volunteers were contacted. However, due to personal reasons one of them could not make it; therefore, 14 people participated. There was a possibility to replace the one who was not available, however, after 14 interviews, a review of the notes taken was made, and the researcher observed that the saturation had been achieved since no new information was emerging. Consequently, the research team decided to stick to 14 participants (non-volunteers). Table 1 provides an overview and details of the study participants.

**Table 1:** Demographic characteristics of the study participants.

Variables	Categories	Volunteers n (%)	Non Volunteers n (%)
Age	35 years and below	13 (43)	8 (57)
	Above 35 years	17 (57)	6 (43)
	Total	30 (100)	14 (100)
Gender	Male	15 (50)	6 (43)
	Female	15 (50)	8 (57)
	Total	30 (100)	14 (100)
Education	None	3 (10)	1 (7)
	Partial primary	6(20)	4(28.5)
	Complete primary	15 (50)	4 (28.5)
	Secondary (partial or completed) and above	6 (20)	5 (36)
	Total	30 (100)	(100)

### Data collection instrument

A semi-structured interview guide showed in S1 and S2 Appendices was developed by the researchers based on the research questions. Open-ended questions and probing were used to get an in-depth understanding of motivations and barriers.

The guide was flexible to allow exploration of new ideas presented by the interviewees and to enable the addition of questions and probing as the data collection progressed. The guide had two versions: one for volunteers and another for non-volunteers. The volunteer version was used to explore the motivational factors for making decisions to participate and submit initial citizen science data, for continued participation, as well as for willingness for future participation. It also included questions related to barriers for staying involved, perceived reasons to stop, and perceived barriers for future participation. The non-volunteer version included the questions related to barriers to get involved in the CSP and willingness to get involved. Table 2 provides details about the components of the interview guide.

**Table 2:** Components of the interview guide.

Study participants	Main themes	Phases	Main interview questions
Volunteers	Motivation	Initial	After the participatory design workshop, you have decided to join the program, what were your reasons for joining this program?
		Ongoing	We started this program in November, and until now you are still involved, What are your reasons for continuing your participation in this program?
		Willingness for future participation	Anytime this research can get to an end, but given the benefits of the program, we may decide that it can continue, what do you think about participation in this program after the completion of this research?
	Barriers	Barriers to stay involved	So far, what barriers were faced while participating?
		Reasons to stop participation	What reasons are you considering (would make you leave the project) to stop?
		Barriers for future participation	What barriers and or challenges could you anticipate from participating in the program after the completion of this research?
Non-volunteers	Barriers	Barriers to get involved	After the participatory design workshop, you have decided not to be part of volunteers, what made you unwilling to join the program?
		Willingness to join the project	Did you had a chance of reflecting on the workshop afterward and felt that you could have taken a different decision? (Explain)

### Data collection procedure

Overall, 44 individual in-depth interviews were conducted to capture participants’ reflections on motivational factors to participate and barriers to get and stay involved in the CSP on malaria control. A list of participants drawn during the participatory design workshops [4] as well as monthly reports from volunteers were used to select the participants to be interviewed. To schedule interviews, participants were contacted via mobile phones, and whoever’s mobile phone was off, he/she was contacted several times, or a representative of the volunteers in the respective village was used (through phone calls) to reach the selected participant. Data were collected in March 2019 (this means after four rounds of monthly citizen science data collection as reporting started in November 2018). The interviews were conductedby the first author who is trained in qualitative data collection. All participants provided verbal consent for being interviewed and recorded. A digital voice recorder was used together with taking notes for each interview. All interviews were conducted at the Ruhuha health center, a central and convenient location for all study participants. The duration of the interviews ranged from 25 to 50 minutes. No repeat interviews were carried out. In addition, transcripts were not returned to participants for comments or corrections. However, some issues raised related to the maintenance of the program were further discussed in a workshop with all volunteers to enhance the retention rate.

### Data analysis

Each interview was recorded and later transcribed. A qualitative content analysis was used [33]. This approach allows for obtaining direct information (codes and categories) from the data analysis without having or imposing predetermined categories [33], hence getting a deeper and richer understanding of the phenomenon under study. After transcription, the demographic data were extracted from Word to an Excel sheet and for reasons of confidentiality, each participant was given a code that was then used in the presentation of the results. For the first round of reading, open coding was performed for ten interviews to become familiar with the data and preliminary codes were identified.

After that, all interview documents were transferred to ATLAS.ti, and open coding was performed to all documents using the identified codes. As indicated by Elo and Kyngas [34], open coding involves writing notes and headings in the text while reading the transcripts. Through coding, verification was also done to check whether there is data that may not fit the identified codes, hence adding new codes; or whether there are codes that may be overlapping or similar in which case they could be merged. From there, the related codes were grouped into categories. The initial set of codes and categories were independently developed by the first author. Another member of the research team (PMP) further independently reviewed the results of this initial set of coding and suggested some changes. No discrepancy was observed from this independent review. The categories were revised and checked to ensure that they were mutually exclusive, thus a final list of categories was made. These categories were then grouped into subthemes.

To explore changes in motivational factors over time (initial and ongoing motivational factors), a network view between categories and related subthemes was created in ATLAS.ti. This was done to visualize linkages between categories and the initial or ongoing phases of participation.

For comparing the motivational factors among age and gender groups, the frequencies of the codes using the code-document table were determined by the independent matching of the motivational factors with age and with gender groups. To equalize the coding density (to take into account the size of the groups), normalization was done in ATLAS.ti. The code-document tables were then exported directly to excel to create figures. An audit trail indicating the main stages of data analysis is included as a supplementary file (S1 Fig).

### Ensuring trustworthiness

To ensure the quality of the data, all elements of trustworthiness: credibility, dependability, confirmability, and authenticity were ensured [34, 35]. (1) To ensure credibility, the researcher conducted the interview herself and field notes were taken to complement the recorded data where necessary, (2) the dependability was assured by a careful selection of study participants with an appropriate sampling technique (purposive sampling method). A detailed description of all steps taken in the methodology and presentations of results is provided as well. (3) To ensure confirmability, the study was guided by the supervisors (authors other than the first author). In addition, as indicated in the data analysis section, through discussion, the first two authors (DA and PMP) developed and agreed on coding framework. A consensus of the research findings was made between the principal researcher (first author) and supervisors. Finally, (4) to ensure authenticity, the study was approved by the Institutional Review Board of the College of Medicine and Health Sciences, the University of Rwanda, and verbal consent was obtained prior to each interview. Confidentiality was ensured by removing all identifiers in data before actual analysis.

### Ethical approval

Ethical approval was granted to the study (Approval Notice: No 414/CMHS/IRB/2017) by the Institutional Review Board of the College of Medicine and Health Sciences, the University of Rwanda.

## Results

We first present the demographic background of the participants, followed by the results pertaining to the three research questions of this study. The presentation of the results follows the structure of Fig 1. We show the results by stages of participation in the CSP for malaria control and for each stage, we indicate the corresponding motivational factors and barriers. In addition, the change of motivational factors over time and comparison among age and gender groups are also presented.

### Demographic characteristics of the participants

Overall, forty-four participants were involved in this study. More than half (57%) of volunteers were aged above 35 years and (57%) of non-volunteers were aged 35 years and below. There was an equal number of female (50%) and male (50%) volunteers. Among non-volunteers more than half (57%) were female. Regarding the education level, the majority had partial or complete primary school in volunteer (63%) and non-volunteer groups (57%) (see Table 1).

### Motivational factors

The motivational factors were divided in three main categories including (i) factors that influence the decision to participate and initial participation, (ii) factors that influence retention or continued participating, and (iii) factors that may determine participation for the long run of the reporting activities even beyond the completion of the current research project. The decision to participate and initial participation were combined because there was a high level of participation since the project started. All participants that decided to participate submitted the first observations as part of the CSP for malaria control. In addition, almost all continued in the first year of the project.

### The decision to participate and initial participation

It was clear that people had more than one motivational factor when they decided to participate. Four main factors emerged: (i) curiosity, (ii) desire to learn new things, (iii) helping others, and (iv) contributing to malaria control.

#### Curiosity

Generally, half of volunteers (16/30) decided to participate because they were curious to catch mosquitoes and were interested to use the handmade trap as one mentioned: *“when started I had the curiosity to collect mosquitoes*. *I could not imagine the water bottle catching mosquitoes*! *Until when we started and I was able to collect mosquitoes that is when I believed that it is possible*.*”* (KRPV_AF1). Surprisingly, many people reported that they did not sleep when they first set the trap (for the first month) because they wanted to observe how the mosquitoes are caught by the trap: *“When I started*, *the first month I did not collect mosquitoes and I reported no mosquitoes caught*. *I was very angry and disappointed*. *[*….*] for the following month*, *I was awake the whole night*.*”(YRPV_YF1)*.

#### Desire to learn new things

Some participants (11/30) saw this project as an opportunity to expand their knowledge about malaria and its control: *“The main reason I decided to participate in this project is what I learned when I came for the first workshop*. *[*….*] when I attend a workshop*, *there are always important things that I gain which in turn may help me to control malaria*.*” (BRPV_YF4)*. Participants were interested to see the malaria mosquitoes and thought that through providing feedback and dissemination workshops they will acquire the knowledge and skills about mosquito identification. Some of them were even ambitious that after gaining knowledge on different mosquito species they can easily identify them whenever they see them: *“[*….*]*. *I would not know how a female mosquito looks like*. *Therefore*, *after some months*, *if you will bring these mosquitoes and show us those species*, *I am sure I will gain some knowledge in that*. *[*….*] if I will go in the bushes and see a mosquito*, *I will be able to confidently [*….*] identify the name*.*” (MRPV_YM5)*

#### Helping others

Some participants (10/30) expressed that they were willing to help researchers at the beginning of the program and they wanted to collaborate by collecting and reporting observations as one expressed: *“After the workshop [*….*] I thought that I do not have to look at my interest only and think that there is no payment*. *[*….*] I thought that if researchers are requesting for help to collect and report information without any payment*, *then it would not be right if there is nobody to help them*.*” (BRPV_AF6)*. In addition, one participant already saw the researcher collecting mosquitoes in an area close to his home, and thought the work was tiresome. Consequently, he thought that participation would make the work easier: *“You see that your colleague used to come down there to collect mosquitoes in our village*, *it was tiresome for her*. *Thus*, *after the participatory workshop*, *I immediately thought that I have to participate and make her work easier*. *I wanted to help her so that she will not be tired*.*” (ZRPV_YM2)* Some participants thought that after attending the workshop and gaining knowledge, they would be able to confidently advice their neighbors about the use of malaria control measures: *“[*….*] I can explain and share knowledge with others*, *I mean those who did not have the opportunity to attend the workshop*.*” (MRPV_YF7)*

#### Contribute to malaria control

The majority of the interviewees (22/30) indicated a desire to contribute to malaria control through collecting mosquitoes as they disturb them while sleeping as one stated: “*Right after mentioning that you wanted the volunteers to collect mosquitoes*, *I immediately said that I am going to write my name on the list of mosquito collectors so that I can really catch them during the night because they bite me a lot [*….*]*.*” (MRPV_AF4)*. Other few participants expressed their interest to participate in malaria reduction as they indicated the severity of the disease and its associated consequences. One participant said: *“I always have one goal in my life [*….*] to improve the wellbeing of every Rwandan*. *Normally*, *there are some values that everybody should follow [*….*]*. *I feel that whichever way can be used to control malaria*, *I am willing to go for that and do it*. *I am committed to help the country to eliminate malaria in any way*. *That is the main reason that I did not hesitate to write my name on the list of volunteers [*….*] malaria is a very serious disease that has many consequences to all Rwandan population [*….*]*.*” (BRPV_AM3)*. In addition, some volunteers (6/30) consider participation as a social responsibility to improve the wellbeing of people around them: *“[*….*] I felt that participation will benefit the country*, *and I also benefit when I live with people who have a better health*. *[*….*] thus*, *I always feel responsible to play a role in others’ development*, *better health*, *and better surrounding environment*.*” (KRPV_AM2)*

### Ongoing participation

After deciding to participate, all volunteers collected and submitted the first data and continued to report for the period of the project (one year). Six factors were reported (i) opportunity for learning, (ii) helping researchers, (iii) malaria control, (iv) ease of use of the tools, (v) usefulness of the project, and (vi) recognition.

#### Opportunity for learning

As the program continued, majority of participants (22/30) expressed their interest to learn new things, and this motivated them to continue reporting. This learning process mainly happens during workshops: *“Nothing pushed me to continue except [*….*] gaining knowledge*. *I think if I continue participating*, *obviously I will learn a lot of things [*….*]*.*” (BRPV_YM2)*. Another participant expressed: *“The reason I continued is that although you have explained many things to us*, *for example*, *different measures to be used for malaria control*, *there is still more that we do not know yet*. *I can not specify exactly what it is*, *but I believe there is more to come and I have to make sure that I participate [*….*]*.*” (MRPV_YF7)*.

#### Helping researchers

Some interviewees (8/30) reported being motived to continue because the researchers still need them in the program and they already agreed to provide support. Therefore, they feel obliged to continue in a team of volunteers: *“When you start something*, *you need to continue until the end*. *[*….*] that is the main reason I would put much effort to continue*. *I have to be on the same page as others and continue until the research will end*. *So that by the time it ends*, *I will be confident that I have contributed from the start to the end*.*” (MRPV_YM5)*. Furthermore, respondents mentioned that the research was still ongoing, and believed that the results are not obtained: *“The reason I have to continue collecting and reporting the observations is that there is no way the research can continue if we do not submit the observations*. *You cannot say that you are doing malaria-related research with a focus on mosquitoes when you do not have those mosquitoes*. *[*….*] thus*, *the research can only achieve its objectives*, *when you have those mosquitoes*, *and we are the one to collect and submit them*.*” (MRPV_YF7)*

#### Malaria control

Some respondents (7/30) indicated that they were interested to continue because the research was related to malaria. By submitting the observations, the results can show a picture on the mosquito density in their villages, and this can indicate a correlation with malaria risk as well: *“I continued reporting the observations because there is a time you can collect one mosquito this month*, *and probably next month you see an increase in number of mosquitoes caught*. *When increased*, *that will indicate that there is also an increase in malaria risk*.*” (MRPV_AM8)*. Furthermore, participants indicated that it is the responsibility of every citizen to play a role to control mosquitoes and malaria in general. In this regard, researchers were seen as stakeholders in this control action: *“I always believe that our government is doing a lot in terms of mobilizing people to control mosquitoes*. *In this regard*, *researchers came also as important stakeholders so that we can play a large role in fighting against mosquitoes*.*” (ZRPV_YM6)*

#### Ease of use of reporting materials

The majority of the respondents (20/30) indicated that participation (collecting mosquitoes through setting up a handmade trap) did not require effort and this, in turn, had an effect on the retention of the participants. Some expressed that waking up during the night to change the batteries of the torches is not a problem as they also generally wake up at night and move around the compound for the security of their domestic animals: *“Personally*, *that time of setting up the trap and follow it up to change the batteries of the torches during night is not a problem*. *Furthermore*, *in a normal circumstance*, *as a man in the house*, *I usually wake up during the night and go out to see what is happening in the compound especially when you have cows or other domestic animals*.*” (MRPV_AM1)*

#### The perceived usefulness of the program

The usefulness of the program was key (25/30) in maintaining volunteers’ interest to continue participating. Some respondents indicated an increase in using malaria control measures and being able to mobilize their neighbours about malaria control. As a result, some interviewees reported a perceived decrease in malaria cases in their families and getting positive feedback from their neighbours:*”The most important reason that motivated me to continue is the effect of these activities in my family*. *Since I started participation*, *I am using malaria control measures*, *and consequently*, *I have observed a reduction in malaria cases [*….*]*, *I always mobilize my neighbours*, *and they have been telling me that the advice given was helpful [*….*]*.*” (ZRPV_YM5)*. Other participants continued participating because they expect more benefits in the future. Some participants believed that once researchers get the results, they will provide the information back to them, and try to solve the problem in collaboration with the policymakers: *“After reporting the observations and having the information about the results*, *you will give us advices*, *or do something to resolve the problem*. *[*….*] if I do not participate and provide the report*, *there is no way that the researchers or policymakers can be able to know the target location [*….*]*.*” (BRPV_YM2)*

#### Recognition, attribution, and expectations

Some interviewees expressed that they do not expect anything in return as a payment (monetary incentives). However, most of them (28/30) indicated a need for recognition. Participants reported a range of minimal recognition that could motivate them to continue reporting and facilitate long-term participation. These include (i) feedback provision, (ii) visits, (iii) more workshops, and (iv) ticket reimbursement for attending a workshop. In relation to the feedback, participants stated: *“When we receive that Short Message Service (SMS)*, *we see that you always think about us and our effort*. *Because giving feedback means that we are still together in the program [*….*] and you value the work that we are doing*. *This is encouraging*.*” (BRPV_YM2)*

Some interviewees indicated a need for more interaction with researchers during village visits, home visits, or phone calls: *“Village visits and interaction with researchers would motivate people to continue reporting*. *Because if you visit people in their villages*, *they*, *in turn*, *think that you care about them and value what they are doing*. *For example*, *yesterday when you called me for this interview*, *I really felt very happy because you still remember me*. *Thus*, *you can plan village visits*, *or phone calls because it is also an interaction*. *You can even plan some home visits*, *or visit us in our isibo meetings*.*” (KRPV_AM2)*

Furthermore, some participants expressed a need for more workshops and expressed ticket reimbursement as one of the motivating factors. This was expressed thinking that when they attend a workshop they usually spend the whole day without doing any other activities: *“You may observe that it has been a long time without having a workshop for all volunteers*, *and you may invite all of them together*. *After the workshop*, *you may say that as a reimbursement of the day (because they may not have worked for that entire day) I am giving you this ticket*. *I believe this can motivate us*.*” (MRPV_AM8)*. Some participants also voiced that it would be good for the volunteers to have a common distinctive symbol for their identification: *“I think you can give us a distinctive symbol so that when somebody comes in the meeting or workshop*, *others may know who he/she is and where the person is going*, *they will say that “this person belongs to that group of volunteers”*. *For example*, *you can give us T-shirts with a logo of the project*.*” (MRPV_AF2)*

### Future participation

#### Among volunteers

All volunteers (30/30) indicated a willingness to continue to participate even after the completion of the current research project. Participants indicated that they would like to continue participating so that they will know how (to what extent) mosquitoes are in their home environment given that participation does not require many resources and effort: *“Completing the research project does not mean eliminating malaria*. *Therefore*, *if there is somewhere to submit the report*, *then I can continue [*….*]*.*” (MRPV_AM8)*

#### Among non-volunteers [interest to get involved]

When we asked non-volunteers if they have thought about the project afterwards and may be willing to participate and change their decision, all of them (14/14) indicated their desire to do so. Some of them felt that it was a failure not to be among volunteers and felt they could have taken a different decision. In addition, few of them visited the volunteers to see how they have been collecting mosquitoes: *“When others started collecting data*, *and I was not among them*, *I thought it was a failure as I took a wrong decision [*….*]*. *I even went to one of the volunteers to see how he collects mosquitoes [*….*]*. *Thus*, *in case there is another opportunity to include other people*, *I am willing to participate*.*” (KRPNV_YM3)*.

Although many non-volunteers indicated their willingness to participate, the volunteers indicated that some non-volunteers think that they are getting paid, and that could be one of the reasons why they also want to be involved: *“There are several people who are willing to participate in my village*, *but some of these think that we are getting paid*. *However*, *there is another category of people who want to know anything that can eliminate malaria in their families*. *Consequently*, *they are willing to do anything possible that can eliminate mosquitoes in their homes*.*” (KRPV_AF1)*

### Barriers to participate in the malaria-related CSP

Several factors were identified that can impede people to get or stay involved in the malaria-related CSP. These barriers were grouped into four: (i) barriers to get involved, (ii) barriers to stay involved, (iii) perceived reasons to stop, and (iv) anticipated barriers for future participation.

### Barriers to get involved

Most of the interviewees (10/14) (non-volunteers) indicated that they were not well informed about the recruitment process that was used. In addition, since they have participated in the participatory design workshops, they thought that this would put them automatically in volunteer groups. Thus, they thought that they would be called back to pick up the materials to be used for reporting the observations: *“I do not know how it happened*. *I remember writing my name on one sheet of paper*, *so I did not follow what was happening on the other sheet*. *For the second time*, *when you distributed the materials and I was not invited*, *that is when I realized that I was not included on the second list of volunteers*.*” (MRPNV_YM1)*

Although the majority stated that they were not well-informed, a small number of participants (3/14) indicated lack of time as a primary reason for lack of participation, and one participant stated that she would not be available by the time of collecting observations because she was planning to be out of the study area for quite some time: *“I was doing some school training in Kigali*, *and I thought if I write my names on the list*, *you may call and find that I am not there*. *Or it would be difficult if I have to travel all the way every month*. *Thus*, *I just took a decision not to participate*.*” (BRPNV_YF2)*. In addition, another indicated that he was too old and thinks that those are the activities for women and youth. Because to him, those are the people that are available most of the time and can closely follow up on those activities: *“I am an old man (68 years old)*, *and I remember I did not write my name on the list of volunteers because I thought the youth will do it*. *I could not imagine at my age collecting mosquitoes [*….*] I have a lot of work to do at home [*….*] and I do not think I can have time to do that*. *There is some work [*….*] for young people*, *those who still have a lot of energy and time[*….*]*.*” (KRPNV_AM1)*

### Barriers to stay involved

The majority of volunteers (19/30) mentioned that they have not encountered any barrier since they started participating. Others mentioned some barriers related to (1) perceived low efficacy of the trap, (2) pressure to collect more mosquitoes, and (3) difficulties related to changing batteries at night.

#### Perceived low efficacy of the trap

The majority of volunteers (17/30) reported low perceived efficacy of the handmade trap that was made available. Some participants were wondering whether the trap has been tested before taking it to the field: *“The challenge is the trap we use while collecting mosquitoes*. *It has been so difficult for me*. *[*….*] mosquitoes are not going inside the trap*. *I sometimes wonder whether you have tested these materials before bringing them to be used in the field*.*” (KRPV_AF5)*

#### Pressure to collect more mosquitoes

Relatedly, as a result of low perceived efficacy of the trap, more than half (16/30) reported pressure to catch mosquitoes, and submitting a report with no mosquitoes was considered as a shame to some of the participants. In this regard, some participants preferred to use other methods (for example catching mosquitoes by only using a net and hands) in case the trap did not catch the mosquitoes: *“I have been considering a report without mosquitoes to be a bad report*. *It does not really look good*. *It would be a problem to submit a report saying that you did not find mosquitoes while they are there [you can hear them making noise]*. *In addition*, *there are many strategies to collect them*. *I do not like to give a bad impression*, *neither I like to withdraw my commitment*. *Thus*, *I preferred to continue and use hands to collect them*.*” (BRPV_AF6)*

Furthermore, instead of using a trap for one day (for one round of collection per month) as indicated, few people preferred to set the trap twice (two days) so that they try their luck and see whether they can collect mosquitoes. For this, some people use the same mixture (sugar, yeast, and water) for two days, or buy other ingredients and make another mixture again: *“For the first time I submitted a report with no mosquitoes*, *but I was really disappointed*. *Then after that*, *I started using two days to see whether I can collect at least a few*. *I could use the same mixture (sugar*, *yeast*, *and water)*. *I had to make sure in the following morning if no mosquito caught*, *I could cover the mixture carefully and use it again in the evening*. *[*….*] I always wish to submit a report with mosquitoes as recommended*.*” (YRPV_YF1)*

However, some respondents (9/30) think they have to report whatever caught by the trap. Thus, they do not feel ashamed when they do not collect mosquitoes as long as they set the trap as recommended. In addition, using other methods to collect mosquitoes was considered by some of the participants to be against the rules of the research: *“[*….*] I think that would be against the rules given from the researchers*. *I always think that there is a reason why you gave us those tools and definitely those are the ones to be used*.*” (KRPV_YF6)*. *“When I do not collect mosquitoes*, *I do not feel ashamed [*….*]*. *What I have to make sure is that I set the trap as recommended*, *that’s it*. *Otherwise*, *if no mosquito caught*, *then it is ok*. *I have just to submit the report*.*” (YRPV_AM2)*

#### Difficulties related to changing batteries at night

A final barrier that was perceived was related to changing the batteries of the torches. Participants reported that sometimes it is difficult to know when the torch gets off, thus a close follow up should be made: *“These days collecting mosquitoes was a challenge*, *due to these tools that we are using*. *It requires a close follow up during the night so that you know when to change the batteries of the torches*.*” (ZRPV_AF4)*. Along the same line, another participant stated: “*Sometimes I wake up at midnight going to change the battery of the torch*, *but I found the torch is already off*, *so in that regard*, *you don’t know exactly when it turns off*.*” (KRPV_AF1)*

### Perceived reasons to stop (leave the project)

All participants believed that there was no reason to stop participating unless they move to another location, get a disease that cannot allow them to continue or is a decision from the researchers: *“I do not think there is a reason why I should stop this activity*. *However*, *today’s world is full of problems and challenges*. *Sometimes you may move to another location to look for other ways of living*, *and these are inevitable*. *Thus*, *if those inevitable reasons may happen*, *I may decide to stop*.*” (BRPV_AM3)*. In the same line, another respondent mentioned: *“As long as I am still alive*, *I will not stop*, *unless I have a chronic disease that may make me a bedridden patient*, *because in this case*, *I would not be able to do it*.*” (KRPV_YF3)*

### Anticipated barriers for future participation

Many participants indicated that there will be no challenge to continue reporting observations after the completion of the research project, as participation does not require effort. However, a majority (20/30) indicated some anticipated barriers including the inability to find materials especially yeast (when they will no longer be offered by the project): *“The main challenge that may even prevent further reporting of observations is lack of materials*. *You see that we use sugar and yeast*, *and not every volunteer may be able to buy them*. *Finding a person willing to buy sugar and yeast for collecting mosquitoes*, *when he/she does not have even a matchbox or soap*, *will be difficult*.*” (ZRPV_YM6)*

### Change in motivational factors

The motivational factors were compared over time (initial vs ongoing), and variations between age and gender were explored.

#### Change of motivation over time

**Fig 2:**
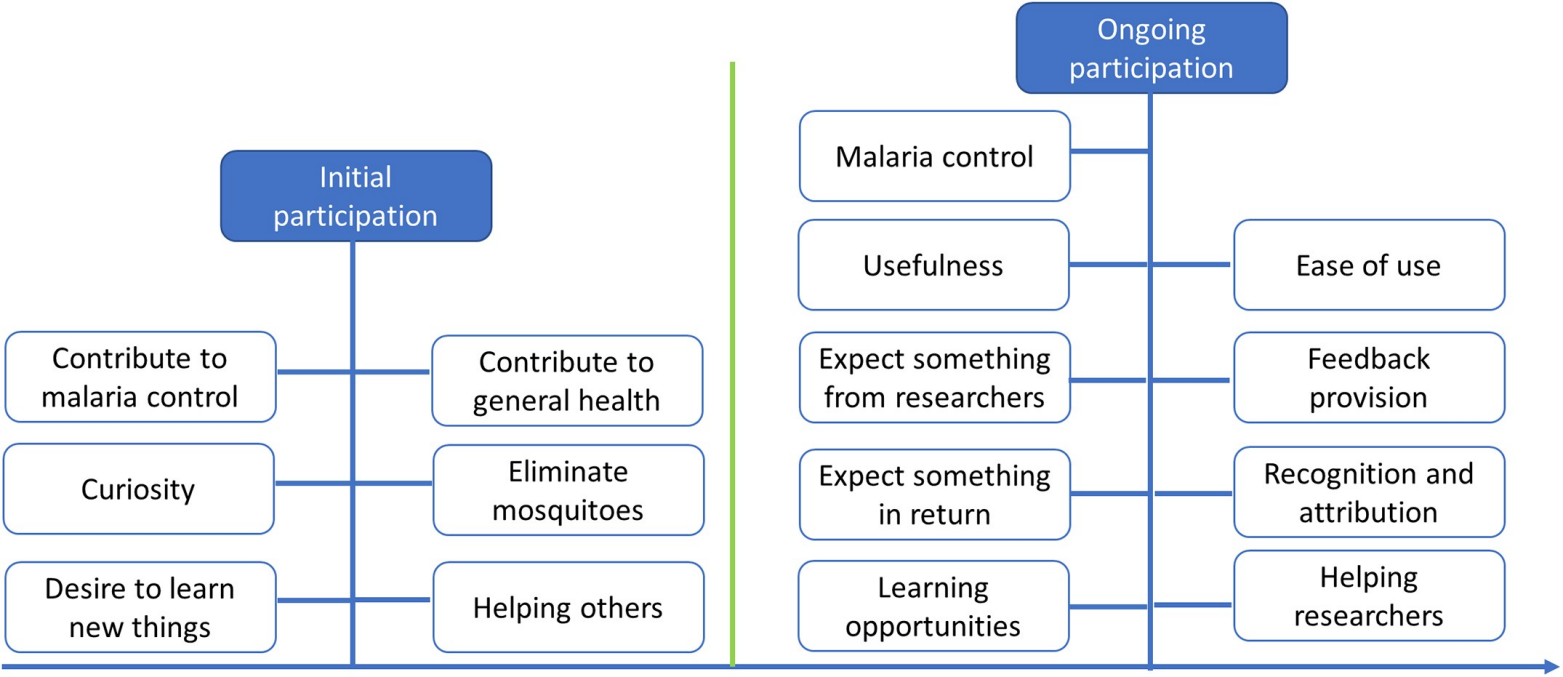
A network view indicating linkages between motivational factors over time (initial vs ongoing participation).

A network view (Fig 2) showed that, when volunteers decided to participate in the malaria-related CSP, they were curious about collecting mosquitoes, wanted to learn new things, and contribute to malaria control. As they started submitting the observations, some motivational factors continued to be present and encouraged them to continue reporting. These included the opportunity for learning, helping researchers, as well as contributing to malaria control. In addition, other factors like ease of use, usefulness, as well as recognition came into play as participation progressed.

#### Comparison of motivational factors by age categories

**Fig 3:**
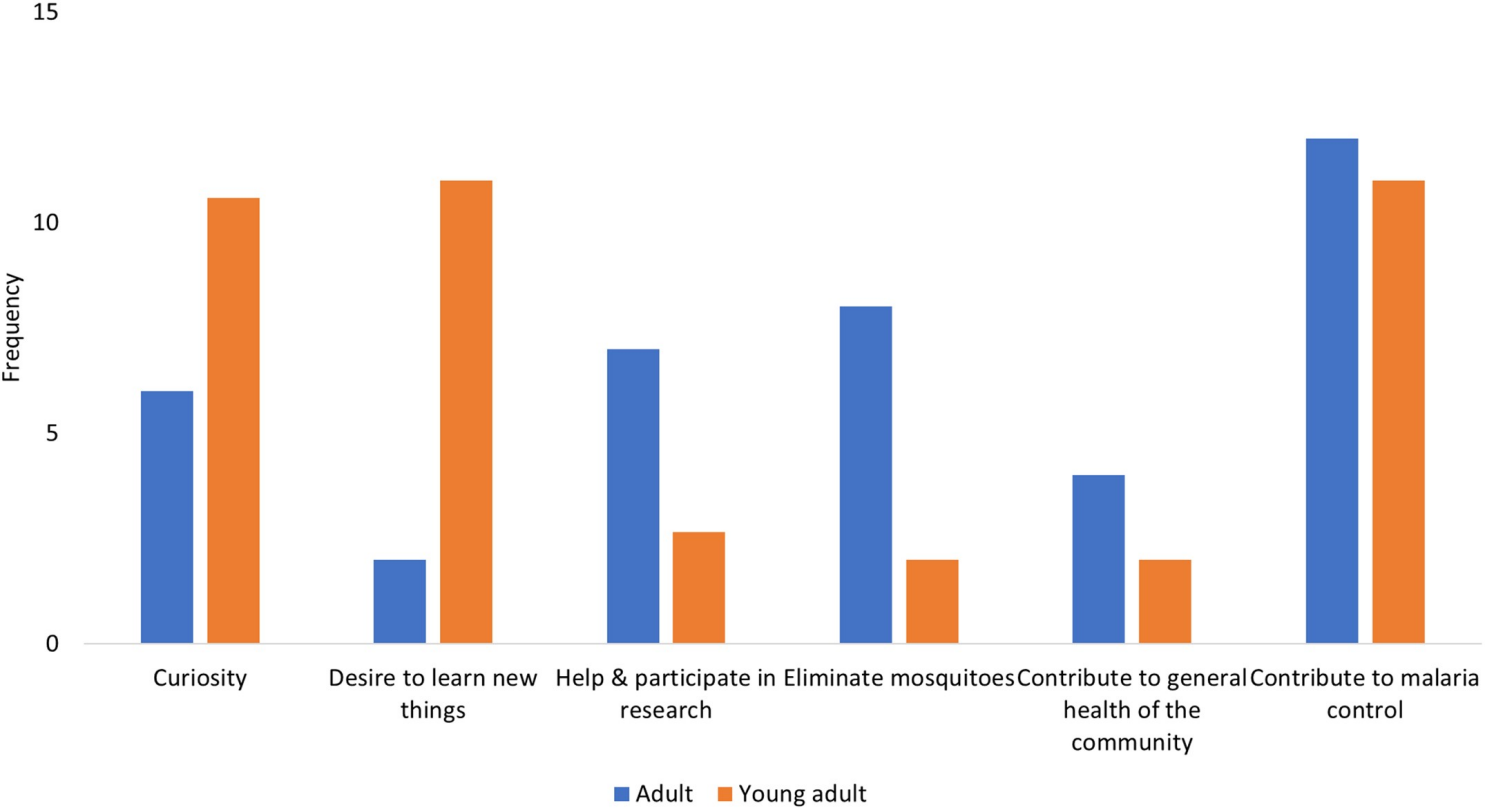
Initial motivational factors according to age.

**Fig 4:**
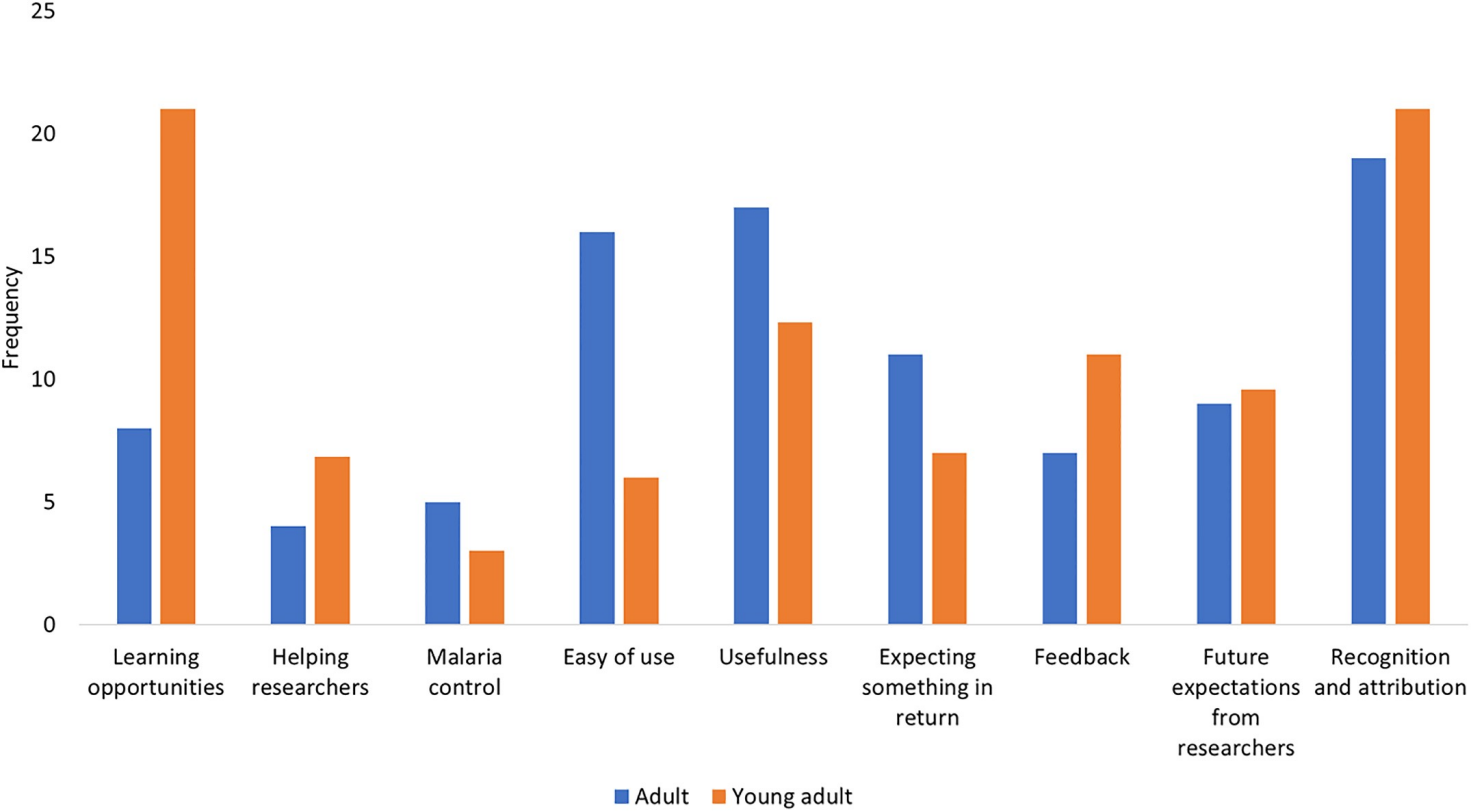
Ongoing motivational factors according to age.

The motivational factors (both initial and ongoing) were compared between young adults and adults (Figs 3 and 4). For initial motivation, these two groups were almost equally motivated to contribute to malaria control. In addition, curiosity and desire to learn new things were also prominent among young people (Fig 3). For the ongoing phase of participation, the two groups were almost equally motivated by the recognition of their effort. In addition, the opportunity for learning was an important factor among young adults while the usefulness of the program and ease of use of the materials were also central for adults (Fig 4).

#### Comparison of motivational factors by gender

**Fig 5:**
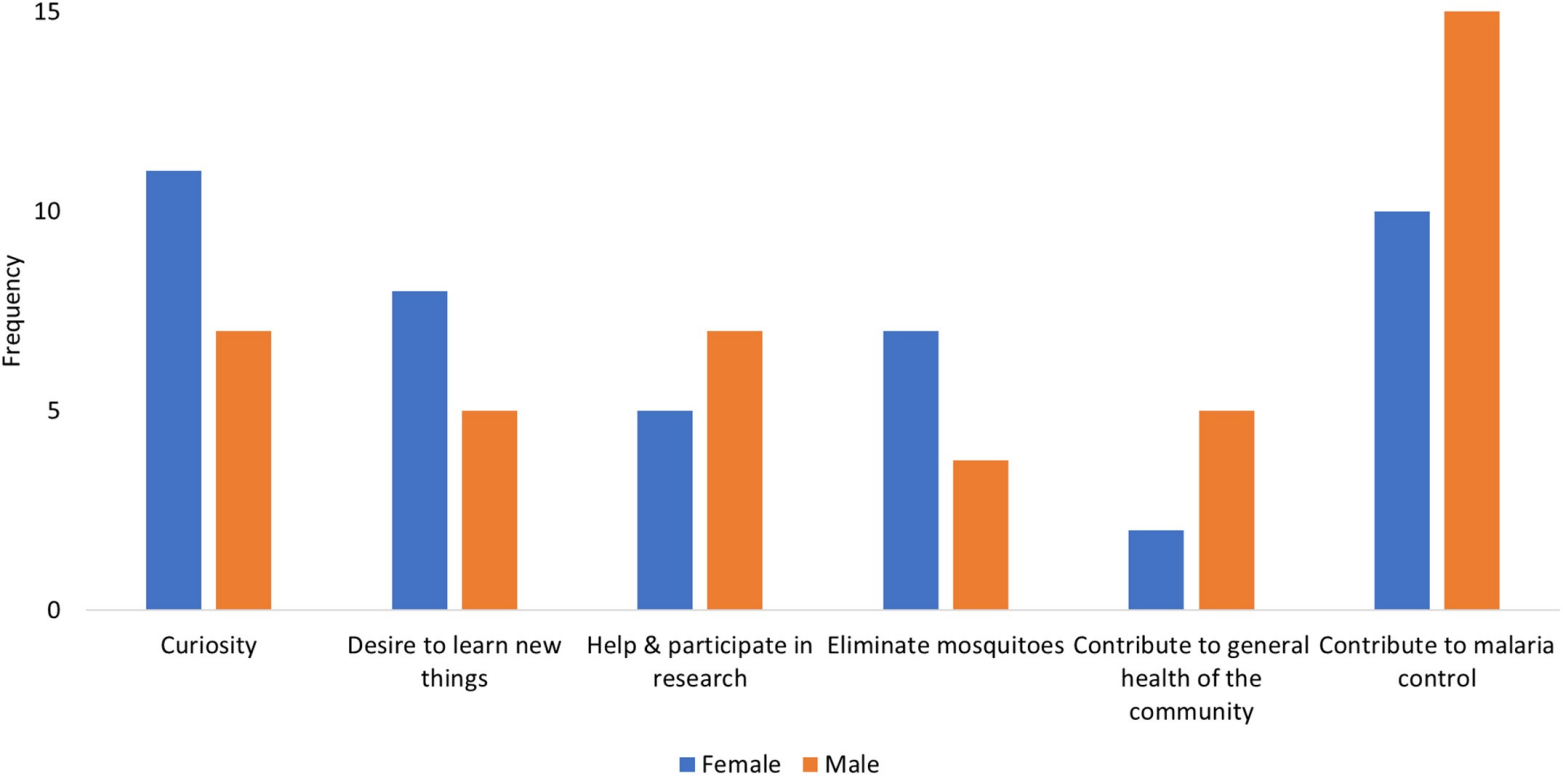
Initial motivational factors according to gender.

**Fig 6:**
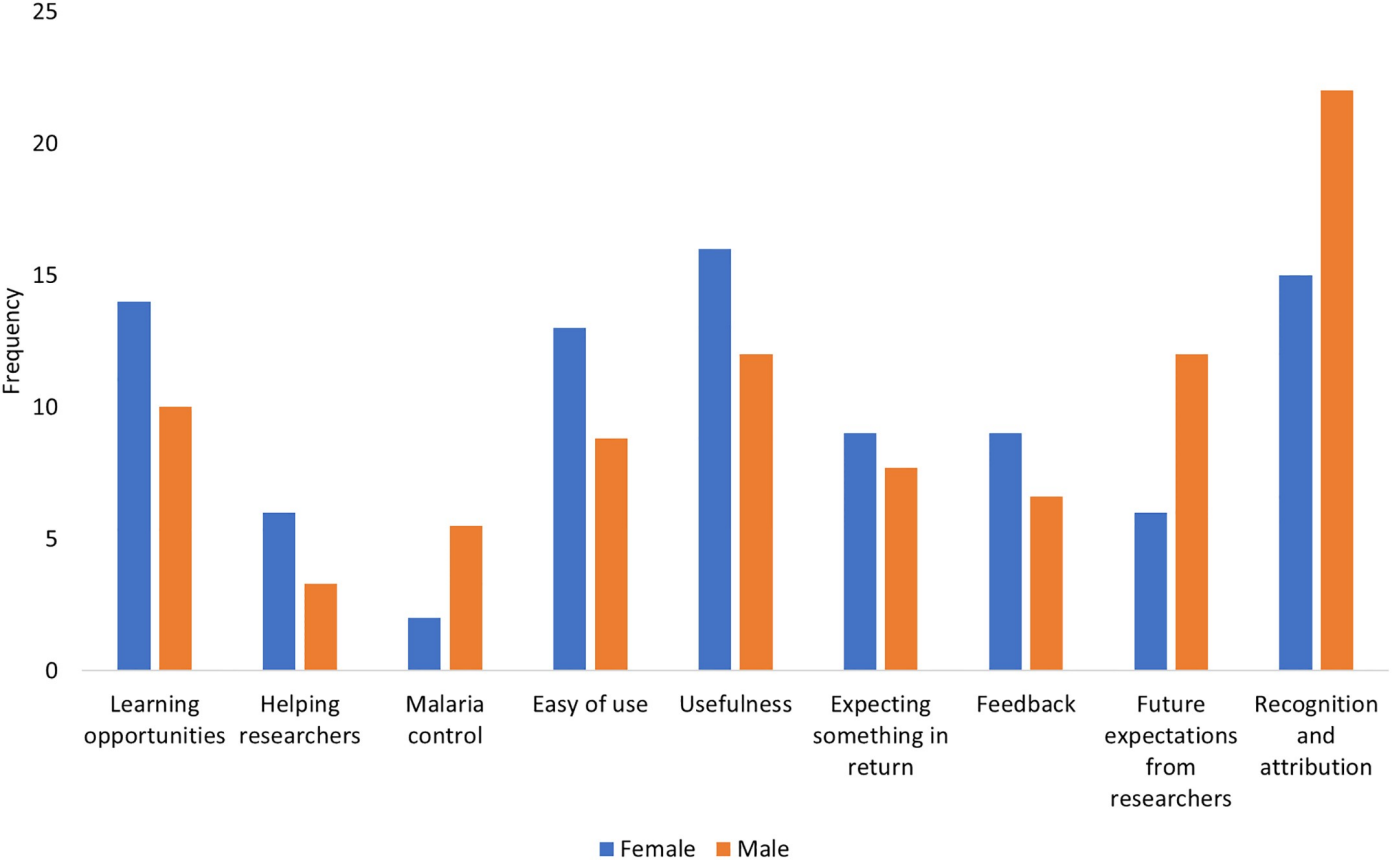
Ongoing motivational factors according to gender.

The motivational factors (both initial and ongoing) were also compared between men and women (Figs 5 and 6). Generally, not many differences were observed between the motivational factors reported by both men and women especially at the initial stage (Fig 5). For both men and women, contribution to malaria control was the most mentioned motivational factor. For women, curiosity was almost equally mentioned as malaria control. For ongoing participation, Fig 6 shows that most women were motivated by the usefulness of the project, ease of use of materials, and learning opportunities, while men were most motivated by recognition of their efforts.

## Discussion

**Fig 7:**
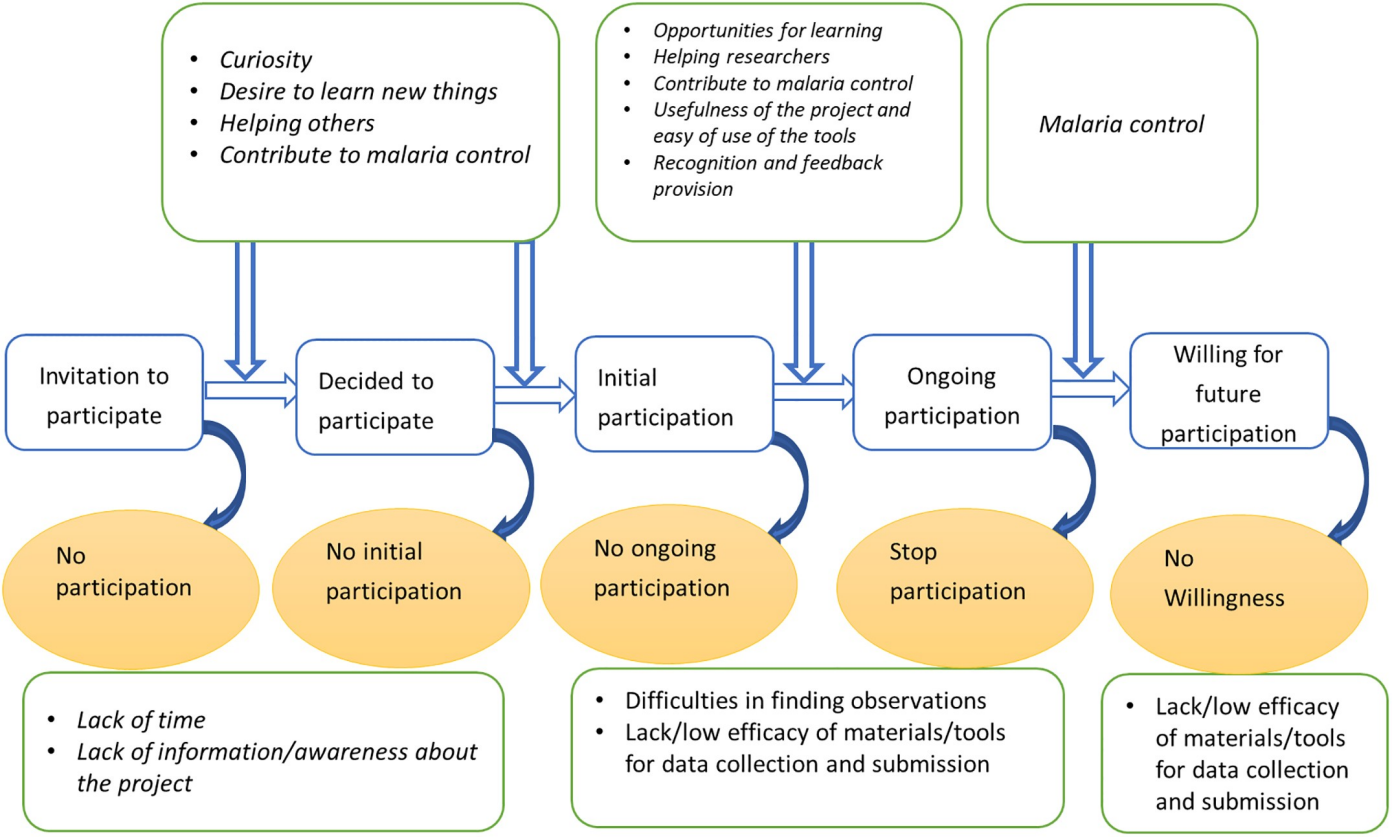
Motivations and barriers to engage in different stages of participation in a citizen science program.

This study explored the motivational factors and barriers to participate in the CSP for malaria control, assessed changes in motivational factors over time, and compared these factors among age and gender groups. The first part of this section discusses the findings (motivational factors, barriers to get and stay involved, and the changes in motivational factors) (Fig 7). The second part presents the implications of the results in a broader context of citizen science. Finally, it presents the strengths, limitations, and avenues for future studies.

### Discussion of the findings

#### Motivational factors to participate in the citizen science program

The findings of this study revealed that at the initial stage, people had different motivational factors including contribute to malaria control, curiosity, and a desire to learn new things. Similarly, some other CSPs also revealed the presence of both personal interest and collective motives at the initial stage like in the Great Pollinator Project [8] and Seeds for Needs project [7]. In contrast with these results, at the initial stage, some other CSPs revealed either the presence of personal factors (egoism) alone, for example in ecological base CSPs [13], or collective factors (value: contribute to health, scientific research, and environment) alone, for example in the iSPEX CSP project [10], the Great influenza project [11], astronomy research [9, 36], and a marine-related project [19].

The presence of both personal and collective factors in the current study may be attributed to the interest in the topic (malaria control) as a means of reminding them to use control measures to protect themselves, their families, and community in general, and interest in collecting mosquitoes as citizen science is a new approach in the study setting. These results corroborate with the findings of great pollinator project [8] which revealed interest in collecting and learning about bees, and contribution to conservation project as top motivational factors for people to join the project. In the same line, Maund et al. [37] revealed contribution to the environment and desire to learn and gain knowledge to be the top motivational factors to contribute to conservation citizen science project. In the current study, the data collection tools may have made volunteers curious to explore how mosquitoes can be caught by the hand-made trap. Equally, the co-design approach that was used may have triggered both motives [4]. The fact that people started participating in the current citizen science program because they wanted to contribute to malaria control is promising. In turn, this may have played a big role in their ongoing participation because they considered malaria as a threat that requires a joint effort for better control.

Opportunities for learning, helping researchers, contributing to malaria control, the usefulness of the project, ease of use of the tools, and recognition were identified as motivational factors for retention. The desire to help researchers to accomplish their tasks has also been reported in the Seeds for Needs project [7]. Sometimes, volunteers help researchers to reach their goals knowing that they will also get something in return [7]. The presence of collective motives in this ongoing stage also corroborates with earlier citizen science studies. For example, a marine-related project, by Carballo-Cárdenas and Tobi [19] found that participants were still concerned about the environment in the later stage of the project, and also other factors including learning, self-enhancement, and socializing came into play. While participating, volunteers have some expectations related to the future use of citizen science data, and this may motivate them to stay in the project. For example, in the present study, volunteers reported that once observations are submitted, the data can be used for further planning about malaria control interventions, hence they were motivated to continue participating. In a similar way, in the iSPEX project, which involves measurements of air quality, volunteers thought that after submitting the measurements, the data can give a clear picture about aerosols in The Netherlands, and the data can have an impact on both health and environmental policies [10], hence this motivated them to join and stay involved in the project. These expectations show how people are concerned about health, issues related to living places and environment, and how these collective motives play an important role in the retention of volunteers [10].

Recognition was mentioned as an important factor that motivated volunteers to continue reporting the observations in the current study. Appreciation of volunteers’ contributions and acknowledgment have been also reported in ecological CSPs to influence the ongoing participation in CSPs [13, 20]. While the majority of volunteers in citizen science do not expect anything in return after sharing the citizen science data, some of these volunteers expect some rewards including information about the topic under study and sometimes monetary incentives [7]. Feedback was considered as one form of recognition. Receiving feedback implies that the work of volunteers is being used for the project’s purpose [13, 38]. The feedback also helps the volunteers to feel part of the project which in turn, affects the engagement in the long term [23].

#### Willingness to participate in future CSP activities

All volunteers were willing to continue participating even after the completion of the research. Previous studies have reported some conditions for future participation in CSPs [10]. These conditions include reminders to report and feedback about the value of volunteers’ contributions. The willingness to participate in future activities depends on the previous fulfillment of individual motivations [26]. As in this study, in the Great Pollinator project, by Domroese and Johnson [8], the appreciation of the project’s activities and willingness to participate among non-volunteers have been reported. This indicates that the feedback provision or sharing project’s activities is an important factor for (1) engagement over a prolonged period and (2) willingness for future participation. When people consider the goals of the project to be a priority in their daily lives, then the willingness to participate or continue participation will increase [39]. The consideration of malaria as a burden in the community where this study has been conducted could have influenced the willingness to participate in the future because through the submission of observations, other malaria control interventions can be implemented in the area.

#### Barriers to get and stay involved in the citizen science program

Lack of time and lack of clear information about the recruitment process were the two most salient barriers among those who did not participate in the citizen science program. Unlike to this study, the time constraint was a barrier among those who ever participated but later dropped in marine-related projects [18, 19], and the Great Pollinator Project [8]. On the other hand, the barriers among those who never participated in a marine-related project included low perceived threat of lionfish, thus collection of related data was perceived to not have value [19]. In the same way, Rotman, Preece [13] reported (perceived) power relations between scientists and citizens to be a barrier for initial contact and the decision to participate. Considering researchers as trained professionals with power and that they are conducting research for their purposes sometimes hinder the initial collaboration between researchers and volunteers and negatively affect the initial decision to participate [13]. These reported factors among non-volunteers are mainly due to the lack of project related information. The relatively long duration (six hours) of the participatory design workshops used to recruit the volunteers in the current study [4], might have affected the participants’ concentration level. Hence, some of them missed out the information about the recruitment process.

Similar to the current study, difficulties in identifying observations, for example, flowers and or bees were reported as barriers for continuing participation in the Great Pollinator Project [8]. Comparing with ICT-based CSPs, some technology-related barriers were reported as challenges to stay engaged and influenced people to leave the project [18, 19]. For example, Martin, Christidis [18] revealed that technological design-related problems (systems which are not user-friendly and which have limited internet connection) can be barriers for volunteers to collect and submit citizen science data. Additionally, failure to recognize and take into account the interest of volunteers may make them think that their motivations are downplayed and can result in leaving the project [13]. Contrary, when the voice of the volunteers is heard, then it is more likely that they can stay involved [40]. For example, in this study waking up at midnight to change the battery of the torches was reported as a barrier. When this was raised, the volunteers alternatively suggested to buy other types of torches with batteries which can last for the whole night. Consequently, this suggestion was considered by the researchers, and new torches were bought. Thus, without discussion, the researchers would not have known whether the torches needed to be replaced. In turn, this could have affected the collection of citizen science data.

#### Comparing motivational factors to participate in a citizen science program across age and gender

Contrary to this study, Land-Zandstra, Devilee [10] found adult volunteers to be more motivated by their contribution to a CSP and concern for health than young adults. While in addition to malaria control young adults were also motivated by curiosity and a desire to learn new things in the current study, in a water quality monitoring project, Alender [41] also revealed the need for career development to be higher among young people than older volunteers. Besides age, motivational factors were also compared among women and men. Contribution to malaria control was the most mentioned motivational factor for both men and women, and also curiosity was almost equally mentioned as malaria control among women. These findings contradict the results of a Galaxy Zoo project, by Raddick, Bracey [9] where women joined the project because of personal enjoyment associated with the project, while men joined because they wanted to contribute to scientific research. The current results also contradict the findings of Land-Zandstra, Devilee [10] where women were more motivated to participate because they were concerned about health, while men were more interested in science and the fun part of the project.

### Implications of the current results in the field of citizen science

Based on the results of this study, the following implications were formulated:

The present results on motivational factors and barriers largely agree with the findings of ICT-based CSPs [8, 13, 19]. This shows that when volunteers are committed to collect and submit citizen science data, the nature of the project (whether ICT or non ICT-based) does not play a large role. What should be considered is how the citizens are recruited, how they are engaged in the design process, what benefits they are receiving, and how their contributions are acknowledged. The motivational factors should be considered because highly motivated volunteers can collect and submit high-quality citizen science data [42]. This indicates that in the absence of technology-related tools, a CSP can be implemented as long as the citizens are motivated to do so.

Nevertheless, two important factors, feedback provision and recognition, need further emphasis in citizen science. In citizen science, volunteers may not directly or explicitly ask for payment or monetary incentives (for example because of shyness), and in turn, they can ask some valuable objects (for example ticket reimbursement in this case). While monetary compensation can be motivating on the one hand, on the other it can also be a barrier for the CSPs with a low budget and may affect their willingness to learn and the quality of data. This is mainly due to that volunteers may be focusing on getting compensation, and consider it as paid work, rather than as a voluntary contribution with commitment. Therefore, scientists in citizen science have to provide clear information about whether there is any form of compensation and recognition planned in the project right at the start of the project. Equally, other benefits (for example opportunity for learning) should be well articulated at the start of a project.

We did not find “social” (socializing, social network, or social interaction) emerging as a prominent motivational factor, a common factor reported in ICT-based CSPs [19, 28]. In CSPs, the interaction happens either physically (mostly in non ICT-based CSPs) through workshops and meetings, or virtually (in ICT-based CSPs) through forums, blogs, and chats’ groups. Although “social” motivation is salient, however, some ICT-based CSPs also reported this to be the least motivational factor [8, 10, 27]. This may be due to that in some CSPs, volunteers do not meet or interact very often, and they are interested in understanding (learning new things) and value (contribution to health and environment).

The variations observed in motivational factors over time, among age and among gender groups, deserve further consideration in CSPs. Some CSPs are conducted for a certain period of time or target a particular group of people, for example, a FeederWatch project by Martin and Greig [43] only target young adults. Other CSPs experience a high attrition rate throughout different stages of participation [1, 21]. Equally, Brouwer and Hessels [44] also revealed a high dropout among younger volunteers, and these had a lower willingness to participate in future related projects. In the current project, drop-out was rarely observed, as there was a high participation rate throughout the study period, and only perceived reasons to stop could be explored. The high participation rate observed in this study may be the result of the co-design process used prior to the implementation of the program, and monthly feedback provided [4]. These findings indicate that special attention should be given to the motivational factors for each group to prevent loss of interest, keep all motivated, and retain them in the project. Thus, it is important for other CSPs especially those that experienced a high dropout rate or used different design approaches, to assess the relationship between these motivational factors and dropout.

### Strengths, limitations, and future research

This study explored the motivational factors at different stages of participation and provided explanations for the increase or attrition of participation in a CSP over time. These results may inform future CSPs on what to focus on at each stage of participation which may, in turn, increase the retention of volunteers on the one hand, and increase the quality and quantity of citizen science data on the other. In addition, these insights can be used to develop strategies on how to organize and manage volunteers in citizen science projects. Therefore, these findings merit consideration during the design and implementation processes of CSPs. The comparisons of motivational factors made among age and gender groups inform future CSPs on which group of people to reach out and how to motivate them at different points in time. The variations reported among age and gender groups can be further tested through quantitative surveys with volunteers in the citizen science program.

Although a small number of volunteers also belong to other groups that are well known in the community (for example community health workers), having this project in the area with some previous research projects brought some ideas that may be different when a similar project is implemented in a different setting. For example in this study, volunteers indicated a need for T-shirts. This idea could have been the result of observing other groups in the community (for example community malaria action teams) wearing T-shirts of that particular project. The project lasted one year, and the results indicated that the motivational factors change over time. It is not clear whether the presented motivational factors at the ongoing phase would remain if the program continues for another year or more. Thus, when such a program is conducted for a long period, periodical exploration of the motivational factors would be necessary to enhance the generalizability of these findings.

The reported barriers to get and stay involved indicate that information about a citizen science opportunity, a detailed description of the recruitment process, as well as the requirements to participate should be clearly communicated before the recruitment of volunteers. In addition, as people reported the trap to catch less or not be able to catch mosquitoes at all, further studies may explore possibilities to compare the collection of mosquitoes using different types of traps versus using eyes and hands; and determine volunteers’ preferences and acceptance of these methods. This is mostly because some volunteers reported that they no longer mixed the ingredients (water, sugar, and yeast) rather they prefer to use hands. This means that some resources (sugar and yeast) may be wasted when the volunteers may take them but never use them, or use them for other purposes.

## Conclusion

This study explored the motivational factors and barriers at different points in the lifetime of a citizen science program on malaria control. At the initial stage, people participated in the CSP because of curiosity, interest to learn new things, helping researchers, and willingness to contribute to malaria control. After the volunteers submitted observations, other motivational factors came into play for maintaining their participation and these include ease of use of materials to report observations, the usefulness of the project, and recognition. Lack of time and information about the recruitment process were reported as barriers to getting involved. Volunteers reported some challenges while participating in the CSP and these included perceived low efficacy of the trap, difficulties in collecting observations which put pressure on them, and challenges related to changing the batteries of the torches during the night. Some variations in the motivational factors were observed across age and gender. For the initial phase of participation, young adults and adults were almost equally motivated to contribute to malaria control. In addition, wanting to learn new things and curiosity were also prominent among young people. For gender, contribution to malaria control was the most mentioned motivational factor for both men and women. In addition, curiosity was also equally mentioned as malaria control among women. For ongoing participation, the young adults and adults were almost equally motivated by the recognition of their efforts. In addition, the opportunity for learning was an important factor among young adults while ease of use of the materials and usefulness of the program were central for adults. With regard to gender, women were more often motivated by the usefulness of the project, ease of use of materials, and learning opportunities, while men were more motivated by recognition of their efforts. This implies that in a CSP, target groups require different recruitment and retention strategies. Thus, future CSPs should consider using different communication channels and strategies at different stages of participation to maximize the recruitment, participation, and retention of volunteers. The desire to contribute to malaria control, both in initial and later stages, is promising. Therefore, as participants were willing to participate for a long period of time, the reported motivational factors need to be considered to retain volunteers in CSPs. The presented motivational framework may be used to explore motivations and barriers in other CSPs.

## Supporting information

S1 Fig An audit trail indicating main stages of data analysis. (DOCX)

S1 Appendix Interview guide (English). (DOCX)

S2 Appendix Urupapuro rw’ ibibazo (Ikinyarwanda). (DOCX)
