# Supplementary figures and images for: Why (not) participate in citizen science? Motivational factors and barriers to participate in a citizen science program for malaria control in Rwanda
Source: PLoS One. 2020 Aug 24;15(8):e0237396. doi: 10.1371/journal.pone.0237396 (PMC7446901; doi:10.1371/journal.pone.0237396)

**An audit trail indicating main stages of data analysis**


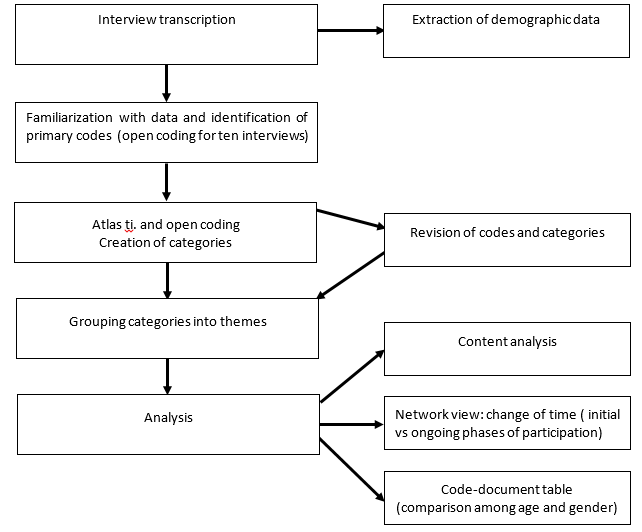

Supplement: S1 Fig — (DOCX) [file pone.0237396.s001.docx]
