# Supplementary material for: Why (not) participate in citizen science? Motivational factors and barriers to participate in a citizen science program for malaria control in Rwanda
Source: PLoS One. 2020 Aug 24;15(8):e0237396. doi: 10.1371/journal.pone.0237396 (PMC7446901; doi:10.1371/journal.pone.0237396)
Supplement: S1 Appendix — (DOCX) [file pone.0237396.s002.docx]

**Appendix 1: Interview guide (English)**

**Part 1: For Volunteers**

Personal characteristics: age, educational level, and employment.

1. You have been participating in this citizen science project, can you tell me a little bit about your experiences from this participation?
2. After the participatory design workshop, you have decided to join the program, what were your reasons for joining this program? (Probes: Do you think that there was one specific reason (or motivation) that mostly affected your decision to participate in this program?)
3. We started this program in November, and until now, you are still involved, what are your reasons for continuing to participate in this program? (Probes: Are there any actions, tools, or reasons that you find especially important in maintaining your interest to participate in this project? How useful is it for being involved? How easy is it for you to use the paper-based form and or collect mosquitoes? Is there anything you expect in return from the researchers as a result of your participation? What and how do you use (could use) the feedback provided by the researchers? How do you think this can be improved? Do you have any other suggestions for us on how people like you can be motivated to stay involved?)
4. Anytime this research can get to an end, but given the benefits of the program, we may decide that it can continue, what do you think about participation in this program after the completion of this research? (Probes: If you are willing to continue, what do you think will motivate you to continue participating after the completion of this research? if not willing, what would make you unwilling to continue participating in this project?)
5. What were barriers were faced while participating?
6. What reasons are you considering (would make you leave the project) to stop?
7. What barriers and or challenges could you anticipate from participating after the completion of this project?

**Part 2: For Non- volunteers**

Personal characteristics: age, educational level, and employment.

1. You participated in the workshop sometimes back (August 2018), can you tell me a little bit about your experiences from this participation?
2. After the participatory design workshop, you have decided not to be part of volunteers, what made you unwilling to join? (Probes: Were there any actions, tools, or reasons that you find especially important for your decision to not participate in this program?)
3. Did you had a chance of reflecting on the workshop afterward and felt that you could have taken a different decision? (Probes: Is there anything we could change or we can do to make you join? If you are asked to participate now, would you be willing to participate in the project (why)? Did you think about this project and probably felt interested after your initial decision of not participating?)
