# Supplementary material for: Why (not) participate in citizen science? Motivational factors and barriers to participate in a citizen science program for malaria control in Rwanda
Source: PLoS One. 2020 Aug 24;15(8):e0237396. doi: 10.1371/journal.pone.0237396 (PMC7446901; doi:10.1371/journal.pone.0237396)
Supplement: S2 Appendix — (DOCX) [file pone.0237396.s003.docx]

**Appendix 2:Urupapuro rw’ ibibazo (Ikinyarwanda)**

**Igice cya mbere: Abakorerabushake**

Umwirondoro w’ubazwa: imyaka, amashuri yize, akazi akora.

1. Umaze iminsi ugira uruhare muri ubu bushakashatsi (muri uyu mushinga), ese wambwira muri make uko wabibonye? Gira icyo umbwiraho?
2. Nyuma y’ amahugurwa twakoze muri Kanama, wemeye kugira uruhare muri ubu bushakashatsi, ese ni mpamvu ki (niyihe mpamvu) yatumye ufata uwo mwanzuro? (Kubaza byimbitse: Ni iyihe mpamvu (impamvu y’ingenzi) yatumye ufata uwo mwanzuro wo kugira uruhare muri ubu ubushakashatsi?
3. Twatangiye mu ugushyingo, kandi kugeza ubu uracyagira uruhare muri ubu bushakashatsi, ese niyihe mpamvu ituma ugikomeje kugira uruhare muri ubu bushakashatsi? (Kubaza byimbitse: Ese nibihe byiza/akahe kamaro byo kugira uruhare muri ubu bushakashatsi? Ubona bimaze iki? Ese ubibona gute (bisaba imbaraga zingana gute, sobanura)? Ese ubona ari ibintu umuntu wese yakora? Ese haba hari ibikorwa, ibikoresho, cg impamvu ubona bifite akamaro kandi bikaba bikomeza ubushake bwawe bwo kugira uruhare muri ubu bushakashatsi? Ese haba hari ikintu waba utekereza ko cyakugarukira/inyungu (igihembo) kivuye kubashakashatsi nk’ igisubizo cyo uko ugira uruhare? Ese ni gute /ni iki ukoresha /ushobora gukoresha ibisubizo tubaha? Ese utekereza ko byakosorwa/byanonosorwa gute ( ibisubizo dutanga, uburyo bibageraho, ndetse nuburyo bikoreshwa? Ese haba hari ikindi gitekerezo watwungura kuburyo abantu nkawe baterwa ubushake kugirango bakomeze bagire uruhare?)
4. Reka tuvuge ko wenda ubu bushakashatsi bugeze kumusozo, ariko wenda bitewe ni ibyiza/inyungu uyu mushinga (ibi bikorwa mukora) bifite, tukavuga tuti umushinga nukomeze, urabyumva gute kuba abantu bakomeza bagakora ibi bikorwa? Sobanura? (Probe: Ese wumva wakomeza kugira uruhare mugutanga amakuru no mugihe ubu bushakashatsi bwaba bwarangiye? Sobanura/ kubera iki?
5. Ese nizihe mbogamizi wahuye nazo muri iki gihe warimo kwitabira ubu bushakashatsi?
6. Ese wumva ari iki cyagutera kudakomeza kugira uruhare muri ubu bushakashatsi? Ese nizihe mpamvu waba wumva zatuma uhagarika kugira uruhare?
7. Ese nizihe mbogamizi watekereza zava mukugira uruhare muri uyu mushinga mu igihe ubushakashatsi bwaba bwarangiye?

**Igice cya kabiri: abatari abakorerabushake**

Umwirondoro w’ubazwa: imyaka, amashuri yize, akazi akora.

1. Wabashije kwitabira amahugurwa yo kuri malariya twagize umwaka ushize mu kwa munani, ese wambwira muri makeya uko wabibonye? Gira icyo umwiraho?
2. Amahugurwa arangiye rero wafashe umwanzuro ko wumva utabasha kugira uruhare muri ubu bushakashatsi, ese ni iki cyaba cyarabiteye? (Kubaza byimbitse: Ese haba hari ibikorwa, ibikoresho, cg impamvu ubona zingenzi zaba zaba zaratumye ufata uwanzuro wo kutagira uruhare?)
3. Ese waba warigeze ugira amahirwe yo gutekereza kuri aya mahugurwa nyuma yuko arangira, hanyuma ukumva wakagombye kuba warafashe umwanzuro utandukanye nuwo wafashe? (Kubaza byimbitse: Ese ubona/wumva hari icyo twahindura cg se twakora cyatuma/kugirango ubasha kugira uruhare? Uramutse usabwe kugira uruhare ubu, ese wumva wagira ubushake bwo kugira uruhare muri ubu bushakashatsi? Kubera iki? Ese waba waratekereje kuri uyu mushinga, wenda ukumva ugize ubushake bwo kugira uruhare nyuma y umwanzuro wawe wambere wo kutagira uruhare muri ubu bushakashatsi?)
